# The Physician’s Visiting List

**Published:** 1898-12

**Authors:** 


THE PHYSICIAN'S VISITING LIST (Lindsay & Blakiston's) FOR 1899. Forty-eighth year of its publication. Philadelphia: P. Blakiston's Son & Co. (successors to Lindsay & Blakiston), 1012 Walnut Street. Price for 25 patients per day or per week, $1.00; 50 patients, $1.25; 50 patients, in two volumes, $2.00; 75 patients, in two volumes, $2.00; 100 patients, in two volumes, $2.25.

This visiting list is the one used by the editor for many years in his daily practice. It is divided by ruled lines into, first, a column for the name of the patient; second, a series of seven narrow columns, one for each of the days of the week, the day of the month being also inserted as a heading; third, a money column for the charge made; fourth, a column for the ledger page, and fifth, a wide column for special memoranda. This is especially useful to homœopathists in noting remedies given, particular symptoms, and other needed notes of the case treated. Additional pages follow for miscellaneous memoranda, a number of pages for noting amounts of accounts asked for; pages for noting obstetric engagements, and finally a general cash account. To this arrangement are added special pages of practical information, such as conversion of metric system of weights and measures into the English system, dose table, comparison of thermometers and table for calculating period of gestation.

